# Scale up of PrEP integrated in public health HIV care clinics: a protocol for a stepped-wedge cluster-randomized rollout in Kenya

**DOI:** 10.1186/s13012-018-0809-7

**Published:** 2018-09-04

**Authors:** Kenneth K. Mugwanya, Elizabeth Irungu, Elizabeth Bukusi, Nelly R. Mugo, Josephine Odoyo, Elizabeth Wamoni, Kenneth Ngure, Jennifer F. Morton, Kathryn Peebles, Sarah Masyuko, Gena Barnabee, Deborah Donnell, Ruanne Barnabas, Jessica Haberer, Gabrielle O’Malley, Jared M. Baeten, for the Partners Scale Up Team

**Affiliations:** 1 Department of Global Health, University of Washington, 325 Ninth Avenue, #HMC 359927, Seattle, WA 98104 USA; 2 Kenya Medical Research Institute, Nairobi, Kenya; 3 Department of Obstetrics and Gynecology, University of Washington, Seattle, USA; 4 Jomo Kenyatta University of Agriculture and Technology, Nairobi, Kenya; 5 National AIDS and STI Control Program Ministry of Health, Nairobi, Kenya; 6 Statistical Center for HIV/AIDS Research and Prevention, Fred Hutchinson Cancer Research Center, Seattle, USA; 7 Departments of Global Health and Medicine, University of Washington, Seattle, USA; 8 Massachusetts General Hospital, Boston, USA; 9 Departments of Global Health, Medicine, and Epidemiology, University of Washington, Seattle, USA

## Abstract

**Background:**

Antiretroviral therapy (ART) for HIV-infected persons and pre-exposure prophylaxis (PrEP) for uninfected persons are extraordinarily effective strategies for HIV prevention. In Africa, the region which shoulders the highest HIV burden, HIV care is principally delivered through public health HIV care clinics, offering an existing platform to incorporate PrEP delivery and maximize ART and PrEP synergies. However, successfully bringing this integrated approach to scale requires an implementation science evaluation in public health settings.

**Methods:**

The Partners Scale Up Project is a prospective, pragmatic implementation evaluation, designed as a stepped-wedge, cluster-randomized trial, operating at 24 clinics in Kenya. In collaboration with the Kenya Ministry of Health, we are catalyzing scaled implementation of PrEP delivery integrated in HIV care clinics. The intervention package includes staff training, clinic streamlined access to PrEP commodity from the Kenya Medical Supply Authority, and ongoing intensive technical assistance to rigorously assess how PrEP delivery is implemented. PrEP service delivery including retention efforts are conducted by the clinic staff with no additional resources from the project. Guided by the RE-AIM (Reach, Effectiveness, Adoption, Implementation, Maintenance) framework and Consolidated Framework for Implementation Science Research, project progress and learning are documented through ongoing monitoring and process evaluations, including chart abstraction and individual and key informant interviews, to evaluate pragmatic rollout and understand barriers and facilitators for successful PrEP delivery in this setting. In this staged rollout design, each step provides data for both pre-implementation (baseline) and implementation periods, and we will compare time points across steps in the baseline versus implementation periods.

**Discussion:**

Cost-effective delivery models are urgently needed to maximize the public health impact of PrEP and ART. The Partners Scale Up Project will set the stage for full-scale PrEP implementation fully run and owned by the Kenya Ministry of Health. The work combines nationally sponsored PrEP delivery with technical support and implementation science from academic partners, defining a new but sustainable paradigm for public health collaboration.

**Trial registration:**

Registered with ClinicalTrials.gov on February 14, 2017:NCT03052010.

## Background

Heterosexual HIV-serodiscordant couples (i.e., in which one member is living with HIV and the other is uninfected) are common in high HIV prevalence African settings [1, 2]. Half of partners of persons living with HIV are uninfected, with women as likely as men to be the HIV-seropositive partner in a serodiscordant couple [3]. Serodiscordant couples are a priority population for implementing HIV prevention interventions, given their high risk [4–6], ability to be identified in generalized epidemic settings through couples HIV testing [5, 7–9], and support within the coupled relationship that facilitates prevention adherence [10, 11]. Pivotal clinical trials demonstrated very high efficacy for HIV prevention strategies in couples [12], including antiretroviral therapy (ART) for HIV-infected partners [13] and pre-exposure prophylaxis (PrEP) for HIV-uninfected partners [12]. A pragmatic, integrated approach to providing ART and PrEP, with ART promoted for the HIV-infected partner and PrEP offered to the uninfected partner until 6 months after ART initiation, or until the HIV-positive partner achieves virologic suppression (a strategy called “PrEP as a bridge to ART”), resulted in near elimination of HIV transmission in a demonstration project among HIV-serodiscordant couples [14].

In September 2015, the World Health Organization (WHO) recommended ART for all HIV-diagnosed persons and PrEP for persons at high risk of HIV acquisition [15]. In July 2016, the Ministry of Health (MOH) in Kenya released guidelines which recommended initiation of ART for all HIV-infected persons irrespective of CD4 count and PrEP for HIV-uninfected persons with substantial ongoing risk of HIV infection [16]. In the guidelines, priority persons for HIV prevention include HIV-uninfected members of serodiscordant couples, particularly those couples in which the HIV-infected partner is not yet taking ART or has not achieved viral suppression on ART.

The Partners Demonstration Project [14] which showed near elimination of HIV transmission using integrated PrEP as a bridge to ART strategy was an important initial step for advancing PrEP and ART for couples from clinical trials to delivery settings. However, translating this success to scale requires effective implementation to achieve public health impact. HIV care in Kenya is principally delivered through public health HIV care clinics, offering an existing platform to incorporate PrEP delivery. Using a stepped-wedge trial design to randomly assign HIV care clinics to scaled initiation of PrEP integrated into HIV care for couples, the RE-AIM (Reach, Effectiveness, Adoption, Implementation, Maintenance) framework [17] and the Consolidated Framework for Implementation Science Research (CFIR) [18] are applied to guide rollout of integrated PrEP for serodiscordant couples in Kenya (The Partners Scale up Project). Here, we describe the design and the application of the RE-AIM and CFIR frameworks for this project.

## Methods

### Goals and specific aims

The overall goal of the Partners Scale Up Project is to serve as a catalyst for expansion of PrEP delivery in public HIV clinics in Kenya and to document best practices for PrEP delivery. The specific aims are:

1. Facilitate delivery of PrEP integrated into ART care for HIV-serodiscordant couples at scale in public HIV care centers in Kenya and evaluate program impact;
2. Assess facilitators and barriers to (a) implementation of PrEP within ART delivery settings and (b) optimized PrEP adherence in public health context with limited measurement capacity;
3. Determine efficiency, cost, and cost-effectiveness of integrated PrEP and ART when delivered in public health clinics; and
4. Develop operational tools that will expand and support delivery of PrEP at scale and secure buy-in from relevant stakeholders to ensure delivery continues to scale up at the national level.

### Design

The Partners Scale Up Project is a prospective, pragmatic implementation evaluation, designed as a stepped-wedge, cluster-randomized trial, operating at 24 clinics in Kenya. In this design, each step provides data for both pre-implementation (baseline) and implementation periods, and data analysis proceeds by comparing time points across steps in baseline versus implementation periods. In collaboration with the Kenya MOH, PrEP delivery was introduced in public health HIV clinics in a staged fashion, with ongoing intensive technical assistance and monitoring and evaluation conducted to rigorously assess how PrEP delivery is being implemented. PrEP provision including behavior risk assessment, demand creation, and retention activities are conducted by public health staff, with no additional resources provided by the project team. Project progress and learning are documented through ongoing monitoring and process evaluations, including chart abstraction and individual and key informant interviews, to evaluate pragmatic rollout and understand barriers and facilitators for successful PrEP delivery in this setting.

### Context

HIV-uninfected individuals with HIV-positive sexual partners became eligible for PrEP per the Kenya national guidelines on use of antiretroviral drugs for treatment and prevention of HIV released in 2016 [16]. A national PrEP implementation framework and service provider toolkit was developed by the Kenya MOH [19], with contribution from the Partners Scale Up Project team. PrEP rollout nationally in Kenya was officially launched in May 2017 and is expanding at a variety of care centers, aimed at providing HIV prevention to key populations [19]. Kenya guidelines specifically recommend PrEP for HIV-uninfected members of HIV-serodiscordant couples until the HIV-infected partner achieves viral suppression with effective ART, in serodiscordant couples desiring safer conception or if otherwise meeting behavioral criteria for individuals to receive PrEP (e.g., additional sexual partners of positive or unknown HIV serostatus, sex work).

### Setting and facility selection

**Fig. 1:**
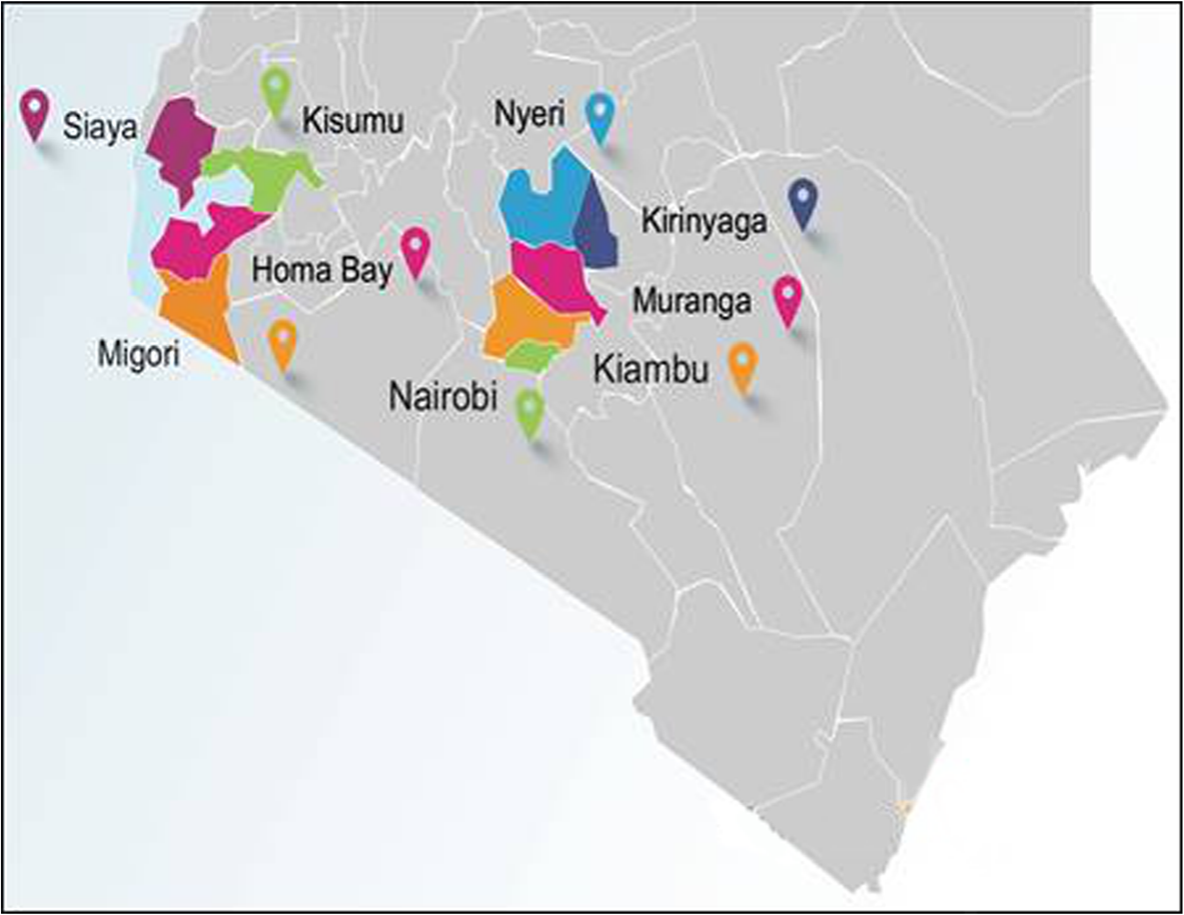
Clinical settings. Counties in Kenya with high and medium HIV incidence

The Partners Scale Up Project is being implemented in 24 Comprehensive Care Clinics (the term used for public health HIV clinics) in two geographical regions of Kenya: Central and Nairobi regions, herein referred to as Central (Kiambu, Kirinyaga, Murang’a, Nairobi, Nyeri counties) and Nyanza, herein referred to as Western (Homa Bay, Kisumu, Migori, Siaya counties). High-volume clinics with established ART programs were identified. Final participating clinics were then selected in consultation with MOH/National AIDS & STI Control Programme (NASCOP) and county health authorities prior to project initiation and reflect the Kenya Ministry of Health HIV priority areas based on HIV prevalence, diversity, and health care settings (Fig. 1). Lessons learnt from the pioneering 24 clinics will be used to inform dissemination of PrEP implementation nationally, including in regions where the project did not initially operate.

### Randomization

**Fig. 2:**
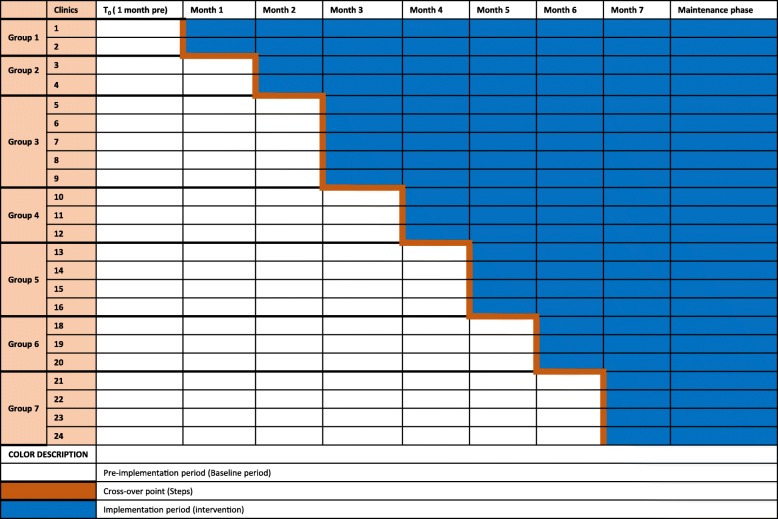
Schema for the cluster-randomized stepped-wedge design

Beginning February 2017, we implemented a stepped-wedge trial design to randomly assign 24 HIV care clinics to scaled implementation of PrEP integrated in HIV clinics for couples. During the project pre-implementation period, PrEP was sanctioned as part of  standard of care by the Kenya national guidelines but there were no systematic approaches in place for implementation in clinical settings. The project intervention is to be a catalytic force to integrate PrEP delivery in public HIV clinics nationally and the intervention package includes staff training, clinic streamlined access to PrEP commodity from Kenya Medical Supply Authority (KEMSA), and ongoing PrEP technical assistance. Prior to randomization, the 24 clinics were stratified by region (12 Central, 12 Western), and each clinic was randomized to the order in which it would start implementing PrEP delivery. The randomization process was conducted at a public event attended by national and county health representatives. Facility leadership picked the order of initiation from an opaque bag containing numbered balls labeled from 1 to 12. Following randomization, the implementation was rolled out in groups of two to four clinics (Fig. 2). Program indicators to measure number of serodiscordant couples newly identified through couples HIV testing, number of HIV-uninfected individuals initiating PrEP, and number of HIV-infected individuals newly initiating ART are obtained monthly from all participating clinics starting 1 month prior to the project initiation.

### Sample size

Using 24 clinics with a baseline period, two to four clinics implementing at each step, and 50 at-risk HIV-uninfected persons initiating PrEP per clinic every 6 months (200 couples on average per site overall, 4800 total), the trial is estimated to have > 90% power to detect a minimum 10% difference in the number of at-risk HIV-uninfected individuals initiating PrEP (defined below as Reach) after implementing the intervention, with a two-sided alpha of 0.05. This difference was conservatively chosen, recognizing that as interventions move to scale their impact is often diminished (a “voltage drop” along successive steps of an implementation cascade [20]).

### Project activities

PrEP has regulatory and normative guidance sanction in Kenya and has been defined as part of standard care, in part based on a series of clinical trials and implementation studies done in Kenyan populations [12, 21–29]. Thus, the intention of the Partners Scale Up Project is to study the process of integrating PrEP services for HIV-serodiscordant couples in HIV clinics and not the PrEP medication itself. PrEP delivery activities are fully conducted by MOH staff in accordance with Kenyan national guidelines; project staff conduct technical assistance and monitoring and evaluation activities to characterize the implementation processes, define facilitators and barriers, and measure uptake, adherence, and costs. The components of the implementation (intervention) package (i.e., activities conducted by the project) include (1) health care provider training on clinical delivery of integrated PrEP and ART for couples, (2) development of operational tools for PrEP delivery, (3) monitoring and evaluation and implementation process evaluation, (4) economic evaluations, and (5) stakeholder and community engagement.

1. *Health care provider training*: Training health care providers is an essential component of implementation of a new clinical strategy in a public health setting. Health providers from each clinic were trained using a 2-day case-based interactive curriculum, developed in conjunction with the Kenya MOH [19]. Content specific to HIV-serodiscordant couples was added, including topics on HIV treatment as prevention and providing couples-based care, with the goal of equipping health providers with the knowledge and skills to provide PrEP to the HIV-uninfected person in a HIV-serodiscordant partnership. A standardized pre- and post-test about PrEP and antiretroviral-based HIV prevention was used to assess knowledge before and after the training. Clinics began PrEP delivery after the training following the randomization order.
2. *Development of PrEP delivery operational tools*: The project has developed operational tools to expand and support delivery of PrEP at scale including a clinical encounter form for documenting PrEP visits in clinics that has been adopted by the Kenya MOH to be the service delivery tool for PrEP implementation for all populations in Kenya (i.e., not only couples and not only at the 24 clinics in this project). The form captures demographic information and ongoing evaluation for behavioral HIV risk factors, HIV testing and other laboratory monitoring, PrEP pharmacy dispensing, adherence self-rating, and side effects.
3. *Monitoring and evaluation and implementation process evaluation*: The Partners Scale Up Project is conducting rigorous process evaluation activities to monitor how effectively PrEP delivery is implemented, including mixed-methods assessments to explore and gain deep understanding of the process of PrEP implementation and contextual influences on implementation and outcomes in clinical settings. Domains defined in the RE-AIM implementation science framework [17] (Table 1) are used to investigate the extent to which PrEP is delivered to at-risk persons (Reach), important clinical or behavioral outcomes (Effectiveness), uptake by clinics and patients and provider acceptance (Adoption), consistency of implementation (Implementation), and contextual factors that influence institutionalization of PrEP delivery over time (Maintenance). Project activities for monitoring and evaluation include ongoing technical assistance, clinical data abstraction, objective measurement of PrEP adherence, and qualitative interviews with PrEP users, health providers, and managers.

**Table 1:** Application of the RE-AIM framework to evaluate the programmatic scale-up of PrEP integrated into public health HIV clinics

Domain	Original RE-AIM definition	Measurement level	Project-specific outcome measures
Reach	Reach is the absolute number, proportion, and representativeness of individuals who are willing to participate in a given initiative	Individual	▪ Number of at-risk persons initiated on PrEP ▪ Demographic and behavioral characteristics of PrEP initiators
		Clinic	▪ Characteristics of implementing clinics ▪ Demand creation strategies ▪ Retention strategies
Effectiveness	The impact of an intervention on outcomes, including potential negative effects, quality of life, and economic outcomes.	Individual	▪ Incident HIV infection among PrEP users ▪ Proportion of random blood samples with detectable tenofovir levels ▪ Frequency of adverse clinical events related to PrEP use
		Program	▪ Cost and cost-effectiveness outcomes: unit cost, HIV infections averted, ICER, DALYS
Adoption	Absolute number, proportion, and representativeness of settings and intervention agents who are willing to initiate a program	Individual	▪ PrEP continuation rates ▪ Barriers/facilitators for PrEP initiation and use
		Clinic	▪ Number of clinics implementing PrEP in HIV clinics ▪ Number of MOH clinical staff trained on delivering PrEP ▪ % of trained MOH staff scoring > 80% on post-test ▪ % of trained clinical staff who delivered PrEP at least once ▪ Internal and external factors influencing PrEP implementation
Implementation	The intervention agents’ fidelity to the various elements of an intervention’s protocol	Clinic	▪ Number and % of users appropriately initiated on PrEP ▪ % of trained staff who delivered PrEP at least once ▪ Clinic innovations and adaptions ▪ Consistency of implementation across staff
Maintenance	The extent to which a program or policy becomes institutionalized or part of the routine organizational practice	Individual	▪ 6-month PrEP continuation rates
		Clinic	▪ Number of clinics implementing PrEP in HIV clinics ▪ Number of clinics that have integrated PrEP delivery in HIV clinics as an on-going part of their regular activities (i.e., have PrEP goals and targets, PrEP in service charter, routine health talks, regular staff training, implementation, PrEP data for monitoring and evaluation) ▪ Internal and external factors influencing PrEP implementation ▪ Number of clinics regularly completing PrEP M & E tools and report PrEP indicators to MOH
		National program	▪ PrEP delivery in HIV clinic continuing as part of Kenya MOH program ▪ M & E tools supplied to clinics ▪ PrEP indicators defined expected

#### PrEP technical assistance

The project conducts health care provider training, as noted above, and then ongoing on-site and remote mentorship. Technical advisors are project staff with expertise in PrEP research and clinical delivery. Each technical advisor oversees up to four clinics and conducts bi-monthly visits to each clinic to coach and mentor the health care providers and observe and document PrEP implementation processes, with a specific focus on processes that are effective or not and aspects of delivery that are changing with time. Using rapid-cycle analysis approaches, technical advisors make summary reports at the end of each clinic visit highlighting what changed, why it changed, who initiated the changes, and outcome of any changes instituted. Key emerging themes that impact PrEP delivery processes are quickly fed back to the facility for possible actions. Best practices for demand creation, clinical PrEP provision, and retention strategies are discussed with and shared across clinics for possible adoption. Detailed clinic-level reports are generated at baseline and 6-monthly to triangulate delivery processes with core components focused on changes in demand creation strategies, clinical PrEP provision, retention strategies, workforce and infrastructure, and monitoring and evaluation.

#### Data abstraction

Clinical records and program data are documented on standardized PrEP delivery MOH monitoring and evaluation systems. Dedicated project staff abstract program data (e.g., number of PrEP initiations, number of ART initiations) and individual clinical records. Individual data is used to characterize PrEP initiation, HIV risk profiles of PrEP initiators, self-reported adherence, retention, HIV infection, and side effects.

#### Evaluation of PrEP adherence

Adherence is essential for PrEP efficacy [22, 30]. At a subset of randomly selected visits (up to 10%), blood samples are collected from individuals taking PrEP and from possible seroconverters to test tenofovir levels as an objective measure of PrEP adherence. Additional evaluation of PrEP use includes pharmacy refill records (i.e., picking up each new PrEP supply) and self-report (e.g., self-rating, captured on the PrEP delivery tool). The goal is to understand both execution (i.e., adherence while used) and persistence (i.e., duration of use).

#### Qualitative interviews and exit surveys to understand delivery

Qualitative interviews are prospectively conducted to rigorously understand delivery, at the level of providers, organization (i.e., clinic), and users. Provider interviews with MOH staff at clinics (nurses, counselors, clinicians, clinic managers) focus on operational delivery to understand acceptance, barriers, facilitators, and opportunities for efficiency. After each interview, short debriefing reports are used to identify emerging themes that affect implementation and those are quickly fed back to clinics prior to formal qualitative analysis. Patient interviews also focus on delivery (e.g., waiting times, visit schedule, visiting HIV treatment centers, confidence in the health system, and community impressions of PrEP), structural (e.g., gender roles), individual (e.g., fertility desire), and dyadic (e.g., sexual negotiation skills, trust, power) factors that may influence PrEP implementation and use. Additional complementary exit surveys with a subset of patients focus on user experiences and satisfaction.

4. *Economic evaluation*: Estimating program impact and cost is necessary to inform public health decision-makers when deciding on HIV treatment and prevention policies and resource allocation. Time and motion studies to follow patients through each PrEP service delivery point are conducted to characterize service delivery models, including appropriate personnel, task shifting, and costs. Program cost for delivery, time spent to deliver PrEP, laboratory costs, and retention activities (e.g., duration and cost of phone calls for appointment reminders) are documented. We also estimate the financial and opportunity cost to PrEP recipients of transportation and time spent at the clinic in exit surveys and time and motion studies.
5. *Stakeholder engagement*: The project conducts relevant stakeholder engagement activities to facilitate buy-in and ensure PrEP delivery continues to scale up at the national level. The project team is part of the Kenya national PrEP Technical Working Group (TWG, which is charged with implementing PrEP for all at-risk populations nationwide) and uses this platform to offer guidance and technical support needed to deliver PrEP at scale on a national level. The project regularly conducts stakeholder meetings attended by representatives from the MOH, NASCOP, National AIDS Control Council, county government officials, other PrEP-implementing partners, clinic managers, and providers from all implementing clinics to review progress.

### Outcome measures and data sources

Key quantitative program implementation outcomes will include (a) Reach, defined as the number of at-risk persons initiating PrEP; (b) Effectiveness, defined as the number of HIV-uninfected on PrEP partners staying HIV-uninfected and adherence to PrEP as measured by tenofovir levels in DBS; and (c) Adoption, measured by PrEP continuation, number of serodiscordant couples newly identified through couples HIV testing. HIV risk profiles of PrEP initiators will be characterized using a validated risk score for HIV-serodiscordant couples [31], which we will use to further predict HIV incidence in the absence of intervention [14]. Monitoring and evaluation will measure patient, provider, and clinic level factors influencing delivery including clinic innovations, adaptations, barriers, and facilitators of PrEP implementation and opportunities for efficiency.

The project uses multiple data sources (Table 2) that will robustly synthesize and triangulate PrEP delivery processes to identify contextually relevant strategies for successful implementation, as well as practical difficulties in adoption, integration, and maintenance to inform wider implementation.

**Table 2:** Data sources

Data source	Description	Purpose
Data abstraction	▪ Data abstracted from clinical delivery tools	▪ Define who is initiating PrEP and whether persons are appropriately put on PrEP
Technical assistance	▪ TA reports prepared at baseline and 6-monthly	▪Document details of the process of adoption and integration of PrEP delivery and track changes in PrEP implementation processes. ▪ Rapid cycle analysis to convey to facilities for quality improvement
Qualitative interviews: user and provider	▪ Purposefully sampled patient and key informants involved in the delivery	▪ Gain deep understanding of process of adoption and integration of PrEP delivery and track changes in PrEP implementation processes.
Time and motion studies	▪ Primary data collection	▪ Economic evaluation
Exit interviews	▪ Random on spot user structured surveys at the end of clinic visit	▪ User experiences and satisfaction
Random blood draw	▪ Dried blood spots collected at ~ 10% visits on persons using PrEP	▪ Objective assessment of PrEP adherence (tenofovir levels), resistance surveillance
Observation	▪ Informal	▪ Track changes in PrEP implementation processes.

### Data management and confidentiality

All abstracted health and patient data are handled in a secure and confidential way through adherence to institutional policies and procedures for securely storing, maintaining, and updating health record information. All health records and patient data are securely stored on password-protected and encrypted servers, and these data will not be released externally except under specific data-sharing agreements. All study results will be presented in aggregate, and no individual patient or provider will be identifiable.

### Analysis

#### Quantitative data

The primary outcome will be the number of at-risk persons initiated on PrEP (Reach). The number initiated on PrEP during baseline period (i.e., pre-implementation period) will be compared with that across the implementation observation periods (exposed or intervention period). Generalized linear mixed models, to estimate the primary intervention effect where both differences observed within steps (between randomized clinics) and changes observed within each clinic as it implements the intervention (within clusters), contribute information. A random effect for each clinic, a fixed effect for each time step, and the fixed intervention effect will be fitted for each time period. Categorical variables will be detailed in tables, and continuous measures will be summarized using means and standard deviations or medians and ranges, as appropriate. *T* tests will be used to detect differences in mean levels of continuous variables and chi-square tests for dichotomous variables.

#### Qualitative data

Qualitative data will be used to interpret and explain the results of the various individual RE-AIM dimensions or patterns of results across dimensions and clinics. We will use an inductive, content analysis approach to analyze the qualitative data (i.e., technical assistance reports, individual and provider interviews, and observation reports) involving an iterative coding process and category construction using qualitative analysis software. The development and analysis of the qualitative research codebook will be guided by the domains and constructs defined in the CFIR [18, 32]. The CFIR will provide contextual information on factors important for the intervention’s acceptability, adoption, and maintenance, as well as quality and fidelity in implementation (Table 3). We will characterize facility-level innovations that improve process and what went well and what was challenging and why, thereby identifying options and contextually appropriate opportunities for efficiency. Qualitative analyses will identify and describe key themes and explore variation within themes. Descriptive content will be inductively assembled to form explanatory accounts.

**Table 3:** Application of Consolidated Framework for Implementation Science Research to the Partners Scale Up Project

CFIR domains/definitions	Respective project-specific codes
1. Innovation characteristics	1. Oral prep for HIV prevention
Innovation Source: Perception of key stakeholders about whether the innovation is externally or internally developed	▪ Ownership of the PrEP program at: National, clinic, and provider level, e.g., provider perception of MOH/NASCOP work vs project.
Adaptability: The degree to which an innovation can be adapted, tailored, refined, or reinvented to meet local needs.	▪ Adaptations—clinic level: PrEP eligibility PrEP baseline lab requirements Visit schedules
Complexity Perceived difficulty of the innovation, reflected by duration, scope, radicalness, disruptiveness, centrality, and intricacy and number of steps required to implement.	▪ Perceived difficulty of delivering PrEP: Perceived ease/complexity of counseling and delivering PrEP: time, labs, steps; challenges counseling about counseling about viral suppression
2. Outer setting	2. External influence of prep implementation
External policy and incentives A broad construct that includes external strategies to spread innovations including policy and regulations (governmental or other central entity), external mandates, recommendations and guidelines, pay-for performance, collaborative, and public or benchmark reporting.	▪ External enablers and policy: MOH policy framework and guidance MOH tools MOH supervision County government involvement
Peer pressure Mimetic or competitive pressure to implement an innovation, typically because most or other key peer or competing organizations have already implemented or are in a bid for a competitive edge.	▪ Clinic-level peer pressure: Motivation/pride from being the first to implement PrEP
3. Inner setting	3. Clinic-level factors
Structural characteristics The social architecture, age, maturity, and size of an organization	▪ Infrastructure and staff: Patient volume, space, staffing
Relative priority Individuals’ shared perception of the importance of the implementation within the organization	▪ Clinic-level priority: Tension between PrEP implementation (healthy person) vs ART services at the clinic (sick person)
Leadership engagement Commitment, involvement, and accountability of leaders and managers with the implementation of the innovation.	▪ Leadership engagement: Facility/HIV clinic manager involvement in PrEP implementation
Access to knowledge and information	▪ Staff training: Adequacy of training, models of PrEP training
Available resources	
4. Characteristics of individuals	4. Health care provider factors
Knowledge and beliefs about the innovation Individuals’ attitudes toward and value placed on the innovation, as well as familiarity with facts, truths, and principles related to the innovation.	▪ Provider adoption and experience: Knowledge and attitudes about PrEP
Self-efficacy Individual belief in their own capabilities to execute courses of action to achieve implementation goals.	▪ Self-efficacy: Staff confidence in counseling and delivering PrEP, staff confidence in counseling about PrEP and condoms, confidence in viral undetectable=no transmission (u=u)
5. Process	5. Prep implementation process
Engaging Attracting and involving appropriate individuals in the implementation and use of the innovation through a combined strategy of social marketing, education, role modeling, training, and other similar activities.	▪ Demand creation strategies: Successful and unsuccessful demand creation strategies ▪ Retention strategies Successful and unsuccessful engagement/retention strategies ▪ Stakeholder engagement
Champions Individuals who dedicate themselves to supporting, and “driving through” an [implementation], overcoming indifference or resistance that the innovation may provoke in an organization	▪ PrEP champions: Types, roles, and how they have emerged, challenges, and how to harness champions
External change agents Individuals who are affiliated with an outside entity who formally influence or facilitate innovation decisions in a desirable direction.	▪ External change agents: Roles and impact of PrEP technical advisors, county/sub county AIDS and STI coordinators involvement, other implementing partners
Execution Carrying out or accomplishing the implementation according to plan	▪ Fidelity, clinic innovations, and adaptations—PrEP provision: Clinical encounter form completeness, same-day PrEP initiation, requirements for baseline creatinine and hepatitis B testing, assessment of acute HIV symptoms, PrEP visit frequency/schedule, quantity of PrEP dispensed, PrEP vs condom disconnect between providers and patients, demand creation, and retention strategies ▪ Demand creation strategies ▪ Opportunities for efficient PrEP delivery
Innovation participants Individuals served by the organization that participate in the innovation, e.g., patients in a prevention program in a hospital.	▪ Patient experiences: Confidence in health system, stigma of coming to an HIV clinic, facilitators and barriers to access to PrEP, benefits/challenges of integrating PrEP in IV clinics, confidence in stopping PrEP when HIV partner achieves viral suppression
Reflecting and evaluating Quantitative and qualitative feedback about the progress and quality of implementation accompanied with regular personal and team debriefing about progress and experience	▪ M & E activities: Best practices, role of technical assistance, rapid cycle analysis, adoption of PrEP delivery, quality improvement activities, M & E measures to assess progress

#### Costs and cost-effectiveness analysis

We will estimate total program cost and cost per person-month of PrEP use from data collected from project expense reports, time and motion observations, and commodity costs obtained from implementing partners. We will characterize efficiency as time per client served, time clients spend at the clinic, or cost per client on PrEP per year. Following WHO guidelines, we will estimate the incremental cost-effectiveness ratio of PrEP delivery integrated into public health HIV care clinics relative to current practice. We will use mathematical models to estimate health outcomes of integrated PrEP delivery beyond the scope of this project, parameterizing the models with PrEP use characteristics (i.e., uptake, retention, and adherence) observed in the Partners Scale up Project and prior results from studies of serodiscordant couples and the literature. Incremental cost-effectiveness ratios will evaluate the incremental cost per (1) HIV infection averted, (2) HIV-related death averted, and (3) HIV-related disability-adjusted life year (DALY) averted by integrated PrEP provision, accounting for both additional costs incurred by the PrEP program and HIV care and treatment costs averted. These analyses will help decision-makers define priorities and allocate resources.

### Protocol amendments

This protocol was registered with ClinicalTrials.gov on February 14, 2017 (NCT03052010). Amendments to the study protocol will require approval from the Scientific and Ethics Review Unit of the Kenya Medical Research Institute and the Human Subjects Division of the University of Washington. Any amendments will be communicated via trial registration updates and reported in any published manuscripts associated with the study as necessary.

### Dissemination plan

The study team for this award is committed to public dissemination of results of clinical trial to local stakeholders in Kenya, the global scientific community, and global policymakers. Dissemination of study results will follow principles of good participatory practice. Results will be published in conference abstracts and peer-reviewed journals. Study results will be disseminated through presentations to global and local stakeholders and policymakers in Kenya, including working in close collaboration with the Kenyan Ministry of Health to help foster immediate translational impact. Authorship eligibility will be determined according to the International Committee of Medical Journal Editors recommendations.

### Project status

The project was activated in February 2017 and implementation is currently ongoing and expected to end in 2021.

## Discussion

In moving from clinical trial evidence to programmatic delivery of new interventions, implementation science evaluations can guide optimization and contextualization of the intervention. For novel HIV prevention interventions, like PrEP, the potential pathway for this transition to implementation has been defined as follows: clinical trials are followed by open label extensions, which provide first access to the product in the trial population, then demonstration projects that “road test” the intervention among individuals who had not been in the clinical trials and using strategies to mimic real-world delivery, and finally product introduction, which leads the intervention to scale through integration into existing health systems [33]. We have systematically followed this pathway in moving PrEP for serodiscordant couples from a clinical trial (the Partners PrEP Study) [12] to an open-label extension (the Partners PrEP Study Open-Label Extension) [21] to demonstration (the Partners Demonstration Project) [14] and now to the current work (Partners Scale Up Project).

The Partners Scale Up Project extends the vast experience in HIV prevention science and PrEP in Kenya to catalyze scaled implementation of PrEP delivery for couples in public health clinical settings. The hallmark of our approach is to set the stage for next steps for full-scale implementation with a sustainable PrEP program that is fully run and owned by the Kenya Ministry of Health. The work combines nationally sponsored PrEP delivery with technical support and implementation science from academic partners, defining a new but sustainable paradigm for public health collaboration. Examples of public health practice outputs from the project will include optimized clinic-level PrEP commodity management, development and dissemination of provider guidelines and delivery tools, and clinician technical support.

Translating effective interventions to routine practice is challenging. Integrating rigorous assessment of implementation strategies aimed at optimizing uptake of novel interventions is critical for understanding why an intervention may work or not work and under what context. We have applied the RE-AIM [34, 35] and CFIR [36, 37] implementation science frameworks to systematically plan, investigate, and document PrEP implementation process, thereby building an evidence base for effective and efficient translation of other effective interventions into routine practice. Mixed-methods evaluation is incorporated that will provide in-depth understanding of the PrEP implementation processes and document best practices and challenges and enablers of effective introduction of PrEP in routine public health HIV clinics. This work will inform implementation of similar interventions elsewhere and facilitate interpretation of intervention outcomes.

This project is using a stepped-wedge pragmatic study design to reconcile the constraints between programmatic implementation and need for rigorous scientific evaluations. The design is especially relevant for proven interventions that cannot ethically be withheld but which cannot realistically be moved to scale simultaneously, which is the situation with PrEP scale up in Kenya. Integrated PrEP and ART for couples is an ideal candidate for a stepped-wedge design across HIV treatment clinics, given its demonstrated effectiveness, opportunity to bring to scale, logistical feasibility of scaling and assessing delivery with each wave of implementation, and utility of scaling within a clinic (i.e., randomizing at the clinic rather than couple level).

In summary, Kenya’s enabling policy environment for PrEP implementation permits a rigorous evaluation of national scale up of PrEP delivery for HIV-serodiscordant couples in public health settings. Findings from this work will inform how to effectively integrate PrEP in HIV clinics and how to efficiently roll out this strategy and achieve national impact.

## Abbreviations

ART: Antiretroviral therapy
CFIR: Consolidated Framework for Implementation Science Research
DALY: Disability-adjusted life year
HIV: Human immunodeficiency virus
KEMSA: Kenya Medical Supply Authority
MOH: Ministry of Health
NASCOP: National AIDS & STI Control Programme
PrEP: Pre-exposure prophylaxis
RE-AIM: Reach, Effectiveness, Adoption, Implementation, Maintenance
TWG: Technical Working Group
WHO: World Health Organization

## References

[1] Piot P, Bartos M, Larson H, Zewdie D, Mane P (2008). Coming to terms with complexity: a call to action for HIV prevention. Lancet.

[2] Chemaitelly H, Cremin I, Shelton J, Hallett TB, Abu-Raddad LJ (2012). Distinct HIV discordancy patterns by epidemic size in stable sexual partnerships in sub-Saharan Africa. Sex Transm Infect.

[3] Eyawo O, de Walque D, Ford N, Gakii G, Lester RT, Mills EJ (2010). HIV status in discordant couples in sub-Saharan Africa: a systematic review and meta-analysis. Lancet Infect Dis.

[4] Celum C, Baeten JM (2013). Serodiscordancy and HIV prevention in sub-Saharan Africa. Lancet.

[5] Grabbe KL, Bunnell R (2010). Reframing HIV prevention in sub-Saharan Africa using couple-centered approaches. JAMA.

[6] Murnane PM, Celum C, Mugo N, Campbell JD, Donnell D, Bukusi E (2013). Efficacy of preexposure prophylaxis for HIV-1 prevention among high-risk heterosexuals: subgroup analyses from a randomized trial. AIDS.

[7] Curran K, Baeten JM, Coates TJ, Kurth A, Mugo NR, Celum C (2012). HIV-1 prevention for HIV-1 serodiscordant couples. Curr HIV/AIDS Rep.

[8] Lingappa JR, Kahle E, Mugo N, Mujugira A, Magaret A, Baeten J (2009). Characteristics of HIV-1 discordant couples enrolled in a trial of HSV-2 suppression to reduce HIV-1 transmission: the Partners Study. PLoS One.

[9] Lingappa JR, Lambdin B, Bukusi EA, Ngure K, Kavuma L, Inambao M (2008). Regional differences in prevalence of HIV-1 discordance in Africa and enrollment of HIV-1 discordant couples into an HIV-1 prevention trial. PLoS One.

[10] Medley A, Baggaley R, Bachanas P, Cohen M, Shaffer N, Lo YR (2013). Maximizing the impact of HIV prevention efforts: interventions for couples. AIDS Care.

[11] Ware NC, Wyatt MA, Haberer JE, Baeten JM, Kintu A, Psaros C (2012). What's love got to do with it? Explaining adherence to oral antiretroviral pre-exposure prophylaxis for HIV-serodiscordant couples. J Acquir Immune Defic Syndr.

[12] Baeten JM, Donnell D, Ndase P, Mugo NR, Campbell JD, Wangisi J (2012). Antiretroviral prophylaxis for HIV prevention in heterosexual men and women. N Engl J Med.

[13] Cohen MS, Chen YQ, McCauley M, Gamble T, Hosseinipour MC, Kumarasamy N (2011). Prevention of HIV-1 infection with early antiretroviral therapy. N Engl J Med.

[14] Baeten JM, Heffron R, Kidoguchi L, Mugo NR, Katabira E, Bukusi EA (2016). Integrated delivery of antiretroviral treatment and pre-exposure prophylaxis to HIV-1–serodiscordant couples: a prospective implementation study in Kenya and Uganda. PLoS Med.

[15] World Health Organization (2015). Guideline on when to start antiretroviral therapy and on pre-exposure prophylaxis for HIV.

[16] Kenya Ministry of Health (2016). Guidelines on the use of antiretroviral drugs for treating and preventing HIV infection in Kenya.

[17] Glasgow RE, Vogt TM, Boles SM (1999). Evaluating the public health impact of health promotion interventions: the RE-AIM framework. Am J Public Health.

[18] Damschroder LJ, Aron DC, Keith RE, Kirsh SR, Alexander JA, Lowery JC (2009). Fostering implementation of health services research findings into practice: a consolidated framework for advancing implementation science. Implement Sci.

[19] Kenya Ministry of Health. Framework for implementation of pre-exposure prophylaxis of HIV in Kenya. 2017.

[20] Glasgow RE, Eckstein ET, Elzarrad MK (2013). Implementation science perspectives and opportunities for HIV/AIDS research: integrating science, practice, and policy. J Acquir Immune Defic Syndr.

[21] Baeten JM, Donnell D, Mugo NR, Ndase P, Thomas KK, Campbell JD (2014). Single-agent tenofovir versus combination emtricitabine plus tenofovir for pre-exposure prophylaxis for HIV-1 acquisition: an update of data from a randomised, double-blind, phase 3 trial. Lancet Infect Dis.

[22] Donnell D, Baeten JM, Bumpus NN, Brantley J, Bangsberg DR, Haberer JE (2014). HIV protective efficacy and correlates of tenofovir blood concentrations in a clinical trial of PrEP for HIV prevention. J Acquir Immune Defic Syndr.

[23] Donnell D, Baeten JM, Kiarie J, Thomas KK, Stevens W, Cohen CR (2010). Heterosexual HIV-1 transmission after initiation of antiretroviral therapy: a prospective cohort analysis. Lancet.

[24] Heffron R, Mugo N, Were E, Kiarie J, Bukusi EA, Mujugira A (2014). Preexposure prophylaxis is efficacious for HIV-1 prevention among women using depot medroxyprogesterone acetate for contraception. AIDS.

[25] Ngure K, Heffron R, Curran K, Vusha S, Ngutu M, Mugo N (2016). I knew I would be safer. Experiences of Kenyan HIV serodiscordant couples soon after pre-exposure prophylaxis (PrEP) initiation. AIDS Patient Care STDs.

[26] Mugwanya K, Baeten J, Celum C, Donnell D, Nickolas T, Mugo N (2016). Low risk of proximal tubular dysfunction associated with emtricitabine-tenofovir disoproxil fumarate preexposure prophylaxis in men and women. J Infect Dis.

[27] Mugwanya KK, Wyatt C, Celum C, Donnell D, Kiarie J, Ronald A (2016). Reversibility of glomerular renal function decline in HIV-uninfected men and women discontinuing emtricitabine-tenofovir disoproxil fumarate pre-exposure prophylaxis. J Acquir Immune Defic Syndr.

[28] Mugwanya KK, Wyatt C, Celum C (2015). Changes in glomerular kidney function among hiv-1–uninfected men and women receiving emtricitabine–tenofovir disoproxil fumarate preexposure prophylaxis: a randomized clinical trial. JAMA Intern Med.

[29] Mugwanya KK, Hendrix CW, Mugo NR, Marzinke M, Katabira ET, Ngure K (2016). Pre-exposure prophylaxis use by breastfeeding HIV-uninfected women: a prospective short-term study of antiretroviral excretion in breast milk and infant absorption. PLoS Med.

[30] Anderson PL, Glidden DV, Liu A, Buchbinder S, Lama JR, Guanira JV (2012). Emtricitabine-tenofovir concentrations and pre-exposure prophylaxis efficacy in men who have sex with men. Sci Transl Med.

[31] Kahle EM, Hughes JP, Lingappa JR, John-Stewart G, Celum C, Nakku-Joloba E (2013). An empiric risk scoring tool for identifying high-risk heterosexual HIV-1-serodiscordant couples for targeted HIV-1 prevention. J Acquir Immune Defic Syndr.

[32] Sherr K, Gimbel S, Rustagi A, Nduati R, Cuembelo F, Farquhar C (2014). Systems analysis and improvement to optimize pMTCT (SAIA): a cluster randomized trial. Implement Sci.

[33] Report AVAC. Research & reality. New York: AVAC; 2013. p. 2013.

[34] Jilcott S, Ammerman A, Sommers J, Glasgow RE (2007). Applying the RE-AIM framework to assess the public health impact of policy change. nn Behav Med.

[35] Stoutenberg M, Galaviz KI, Lobelo F, Joy E, Heath GW, Hutber A (2018). A pragmatic application of the RE-AIM framework for evaluating the implementation of physical activity as a standard of care in health systems. Prev Chronic Dis.

[36] Kirk MA, Kelley C, Yankey N, Birken SA, Abadie B, Damschroder L (2016). A systematic review of the use of the Consolidated Framework for Implementation Research. Implement Sci.

[37] VanDevanter N, Kumar P, Nguyen N, Nguyen L, Nguyen T, Stillman F (2017). Application of the Consolidated Framework for Implementation Research to assess factors that may influence implementation of tobacco use treatment guidelines in the Viet Nam public health care delivery system. Implement Sci.

